# Platelet indices parameters in the new disease activity score of rheumatoid arthritis with ankle involvement: A comparative analytic study

**DOI:** 10.1371/journal.pone.0257200

**Published:** 2021-09-16

**Authors:** Safaa A. A. Khaled, Hamdy F. F. Mahmoud

**Affiliations:** 1 Department of Internal Medicine, Clinical Haematology Unit, Assiut University Hospital/Unit of Bone Marrow Transplantation, South Egypt Cancer Institute, Faculty of Medicine, Assiut University, Egypt; 2 Department of Statistics, Virginia Polytechnic Institute and State University, Blacksburg, VA, United States of America; 3 Department of Statistics, Mathematics and Insurance, Faculty of Commerce, Assiut University, Egypt; Nippon Medical School, JAPAN

## Abstract

Background: Platelet indices (PIs) are platelet parameters that are correlated with platelet activity. Despite being widely available, inexpensive, and feasible; their use in clinical settings is limited. Recently, we developed a new score (EgyDAS), which relies on PIs and assesses disease activity in rheumatoid arthritis (RA). Objectives: This study explored the practicability and validity of EgyDAS in RA with ankle involvement, considering that ankle is neglected in the commonly used DAS28 score. Methods: This comparative case-control study included 2-groups of RA patients, group1 (control): without and group 2: with ankle involvement. Results: Ankle involvement in RA showed no gender or age differences, however, it was associated with higher platelet count, erythrocyte sedimentation rate (ESR), C-reactive protein (CRP), platelet distribution width (PDW), visual analogue scale (VAS), tender joint count (TJC), and lower hemoglobin (Hb) and mean platelet volume (MPV). DAS28 categorized a higher proportion of patients to have high disease activity compared with EgyDAS; moreover, it did not detect those in remission in group 2 patients. Highly significant differences in the 2-scores were observed between the two groups. Further analyses revealed superiority of EgyDAS in assessing disease activity in group 2 patients. Finally, both scores were found correlated together in the study groups. Conclusions: Over or underestimation of RA disease activity could occur when using DAS28. PIs were found correlated with ankle involvement in RA. PIs and EgyDAS are the best tools to assess disease activity in RA patients with ankle involvement. However, the study recommended the use of both scores together.

## Introduction

Platelets are the second most abundant cellular components of the blood after erythrocytes. They play a pivotal role in thrombosis and hemostasis. Pls are platelet parameters nearly similar to Red Blood Cell (RBCs) indices. However, they are not extensively used in clinical practice, like RBCs indices, although they could be easily obtained from the automated blood cell count. PIs included MPV, PDW an index of platelet diversity, Plateletcrit (PCT), and Platelet Large Cell Ratio (P-LCR). Of those MPV and PDW are the most commonly searched and/or used indices [1, 2].

Platelets also play an important role in inflammation; platelet factor 4(PF4) was reported to help attraction of monocytes and their subsequent differentiation into macrophages. Accordingly, the association between platelet indices and the degree of inflammation could be logical. An important example is the reported association between PIs and disease activity in patients with RA, the most common inflammatory joint disease [3–6]. Reduction of MPV was found associated with a severe degree of inflammation; this was further explained by the consumption of platelets at the inflammation sites. On the contrary, PDW was found to increase with increasing activity of inflammation [7–9].

RA is a chronic inflammatory deforming polyarthritis. Assessment of disease activity in RA patients is very crucial to guide treatment options, dosing and duration. So far, numerous scales and disease activity scores have been developed to help tailor treatment for each individual patient. However, DAS28 is the one often used in clinical practice. It relies on tender and swollen joint counts together with VAS and ESR or C-reactive protein [10–14]. Some parameters are subjectively allowing over or underestimation of disease activity. Accordingly, relying on DAS28 alone to guide treatment of RA patients renders patients susceptible to serious drug side effects or disease complications. The association between DAS28 and PIs were also proved [15]. Recently, we developed EgyDAS for assessing disease activity in patients with RA. It relies on inflammatory markers, PDW and MPV, thus it could be used alone or together with DAS28 to overcome the problem of over or underestimation [16].

Another important issue about DAS28 is that it neglects ankle arthritis, although a high proportion of RA patients reported ankle arthritis on presentation [17]. This issue could be addressed by the application of the EgyDAS index either alone or concomitant with DAS28.

This study has two objectives: (1) evaluating the EgyDAS in terms of its applicability on RA patients with ankle involvement, and (2) exploring the validity of EgyDAS to assess disease activity in those patients.

## Patients and methods

The power analysis was done for this study as follows. Our main interest in this study is to study the new score (EgyDAS) and ankle disease. So, using EgyDAS score as our main parameter and at effect size 0.90, significance level 0.05, sample sizes ratio of 1.4 (the patient group to control group), and a target power of 0.90, the sample size for the control group is approximately 23 patients and the sample size of the patient group is 32. The power analysis was done by GPower version 3.1.

The applicability and ability of the new score (EgyDAS) in assessing disease activity in RA patients with ankle involvement was studied using a group of 55 RA patients. They were recruited among those attending the Internal Medicine Department, Assiut University Hospital, Assiut, Egypt, over a period of 6-months from August 2020 to February 2021. This group was of the same ethnic group in whom the EgyDAS score was developed. Accordingly, this group was used to assess the validity of the EgyDAS score in RA patients with ankle arthritis. Their inclusion criteria were 1) age ≥ 18-years 2) New cases meet the ACR-EULAR (American College of Rheumatology; EULAR- European League Against Rheumatism) criteria for RA [18]. The exclusion criteria were 1) presence of other chronic inflammatory or infectious disorder, 2) treatment with anti-platelets, anti-rheumatic drugs, steroids and antico-agulants, 3) blood or blood product transfusion over the past 12-weeks, 4) diabetes mellitus, deep venous thrombosis or other thrombosis, and 5) unilateral ankle involvement.

A data collection sheet was created by the researchers to help organize and facilitate data collection. The sheet composed of 6-sections. 1) demographic data section including age, gender, marital status, residence…etc. 2) main presenting complaint and duration of disease 3) articular manifestations including morning stiffness and its duration, number of swollen and tender joints, presence or absence of ankle arthritis 4) extra-articular manifestations including anorexia, weight loss, lethargy, myalgia, rheumatoid nodules, muscle wasting, cardiovascular manifestations … etc. 5) visual analogue scale, and 6) results of laboratory investigations including CBC, ESR, CRP, and PIs.

Patients were interviewed and clinically examined to collect data, assess the presence or absence of ankle arthritis, collect the number of tender and swollen joints, and asses VAS. The collected data were evacuated in the data collection sheet specified by the patient’s code. Based on the collected data patients were grouped into two groups: group 1 is RA patients without ankle arthritis (control) and group 2 is RA patients with ankle arthritis. Analysis of their complete blood counts (CBCs), ESR and CRP was done as part of routine patient care during follow-up visits. The normal adult reference ranges of the used parameters were as following: MPV 7.6–9.3fL; PDW 9.4%-16.0%, ESR 1–15 mm/hr (for males) and 1–20 (for females), and CRP <10 mg/L. Regarding VAS we used a ruler ranging from 0–10 cm (0–100 mm) where 0 means no pain; 5, moderate pain; and 10, the worst possible pain. Their DAS28 score was calculated using RheumaHelper application, and the new RA score was calculated using the equation below:

$$New\;DAS\;\left(or\;EgyDAS\right)=5.78+0.65\times \ln\left(ESR\right)+0.37\times \ln\left(CRP\right)-\frac{7.47}{\sqrt{PDW}}-3.09\times \ln\left(MPV\right)(16)$$

### Statistical analyses

Analyses were conducted using Minitab Statistical Package® (Version 19, Minitab Inc., developed at Pennsylvania State University, USA), the R statistical software and GraphPad Prism V. 5. Numerical parameters were displayed as mean ± standard error of the mean (SE), while categorical variables were presented as percentages out of the total number. T-test on several parameters is conducted to assess the differences and compare the two study groups: control group who has no ankle disease and patient group who has ankle disease in several parameters (the parameters in the DAS28 equation and EgyDAS equation) to find which parameters have a significant impact on the ankle disease. Levene’s test was used to test equal variances in the two study groups in each parameter to assess the equal variance assumption for the t-test. Pearson’s correlation coefficient was used to measure the correlation between the two disease activity scores, DAS28 and EgyDAS. To compare between the two scores–DAS28 and EgyDAS—in discriminating between the two study groups, several criteria were used: t-test p-value, effect size measures (Cohen’s d, Glass’s delta, and Hedges’ g), and logistic regression results (Odds ratio, Akaike information criterion (AIC), Bayesian information criterion (BIC), and receiver operating characteristic curve (ROC)). Chi-square test is used to study the association between categorical variables. The difference was considered significant when P-value < 0.05, and highly significant if P-value < 0.01.

### Ethical statement

The study complies with the declaration of Helsinki, moreover, it was granted approval by the Research Ethical committee and the Institutional Review Board at the Faculty of Medicine, Assiut University. Patients’ written informed consent was a prerequisite for the study.

## Results

### Hematological parameters including PIs in in RA patients with ankle involvement

In this section, the two study groups (patients’ group: RA patients who have ankle disease, 32 patients, and the control group: RA patients who do not have ankle disease, 23 patients) were compared in many aspects: (1) the demographic and clinical characteristics, and 2) hematological profile of the patients including PIs.

#### Demographic and clinical characteristics of the study groups

After clinical evaluation of the 55 RA patients included in the study, 23 (41.8%) patients have no ankle involvement (group 1, control), and 32(58.2%) patients have ankle involvement (group 2). Table 1 showed demographic differences among the studied groups, it revealed insignificant gender differences. However significant differences were observed regarding residence and marital status where approximately half (46.7%), and 93.8% of patients with ankle involvement lived in urban areas and are married, respectively. Moreover, the table revealed a higher incidence of multiple extra-articular manifestations (EAM) in the group with ankle arthritis.

**Table 1:** Differences in demographic characteristics and extra-articular manifestations (EAM) among rheumatoid arthritis patients for the two study groups: The control group (patients without ankle involvement, n = 23) and ankle involvement group (patients with ankle involvement, n = 32) along with the p-value of the *x*^*2*^- test.

Characteristics	Variable	Group	No. (%)	P-value
**Demographics**	Gender (Female)	Control group	18 (78.3%)	0.789
		Ankle involvement group	26 (81.2%)	
	Residence (Urban)	Control group	3 (13.0%)	0.009**
		Ankle involvement group	14 (46.7%)	
	Marital status (Married)	Control group	18 (78.3%)	0.013*
		Ankle involvement group	30 (93.8%)	
**EAM**	Lethargy	Control group	3 (13.0%)	0.019*
		Ankle involvement group	9 (28.1%)	
	Muscle wasting	Control group	9 (39.1%)	
		Ankle involvement group	6 (18.8%)	
	Weight loss	Control group Ankle	0 (0.00%)	
		involvement group	2 (6.2%)	
	Myalgia	Control group Ankle	3 (13.0%)	
		involvement group	0 (0.00%)	
	Splenomegaly	Control group	3 (13.0%)	
		Ankle involvement group	1 (3.1%)	
	Dyspnea	Control group	3 (13.0%)	
		Ankle involvement group	1 (3.1%)	
	Lethargy, myalgia, lymphadenopathy, nodules, and dyspnea	Control group	0 (0.00%)	
		Ankle involvement group	5 (15.6%)	
	Lethargy, muscle wasting, weight loss, lymphadenopathy, and episcleritis	Control group	0 (0.00%)	
		Ankle involvement group	4 (12.5%)	
	Muscle wasting, anorexia, wt. loss, splenomegaly	Control group	2 (8.7%)	
		Ankle involvement group	3 (9.4%)	
	Splenomegaly and lymphadenopathy	Control group	0 (0.00%)	
		Ankle involvement group	1 (3.1%)	

*Means the difference is significance at 5% and

**means the difference is significance at 1%.

Table 2 shows significantly longer morning stiffness of group 2 patients compared to group1 (P-value = 0.001). As regard age and duration of disease insignificant differences were noted between the two groups.

**Table 2:** Mean ± standard error of the mean (SE) of age, disease characteristics and hematological profiles among the two study groups: The control group (patients without ankle involvement, n = 23) and ankle involvement group (patients with ankle involvement, n = 32) along with the p-value of the independent samples t-test.

Variable	Group	Mean ± SE	P-value
**Age (years)**	Control group	46.30±2.512	0.241
	Ankle involvement group	49.72±1.647	
**Duration of disease (MS)**	Control group	61.13±11.318	0.312
	Ankle involvement group	46.84±8.654	
**Duration of morning stiffness (mins)**	Control group	20.22±7.322	0.001**
	Ankle involvement group	55.31±7.106	
**WBCs x 10** ^**3**^ **/ul**	Control group	5.256±0.3119	0.043*
	Ankle involvement group	6.903±0.6338	
**Hb g/dL**	Control group	11.597±0.4986	0.003**
	Ankle involvement group	9.328±0.5091	
**Pltx 10** ^**3**^ **/ul**	Control group	247.04±24.852	0.002**
	Ankle involvement group	406.81±38.080	
**PCT%**	Control group	0.2957±0.0426	0.615
	Ankle involvement group	0.3338±0.0612	

SD: standard deviation, SE: standard error, MS: months, mines: minutes, WBCs: white blood cells, Hb: hemoglobin, Plt: platelets, PCT: plateletcrite.

*Means the difference is significance at 5% and

**means the difference is significance at 1%.

### Hematological parameters and PIs in RA patients with ankle involvement compared with the control group

Regarding the hematological profile, Table 2 shows that RA patients with ankle involvement have significantly higher platelet count and lower hemoglobin compared to the controls, P-value = 0.002 and 0.003, respectively. However, the two groups were nearly age-matched with insignificant differences in PCT.

### EgyDAS versus DAS28 in RA patients with ankle involvement compared with the controls

In this section we 1) assessed RA disease activity levels using EgyDAS and DAS28 in both groups, 2) investigated the association between EgyDAS and DAS28 parameters and ankle involvement in RA patients, 3) discriminated between the two study groups using EgyDAS and DAS28, and 4) studied the association between the two scores (EgyDAS and DAS28) within each group.

#### RA disease activity levels using EgyDAS and DAS28 in the study groups

**Fig 1:**
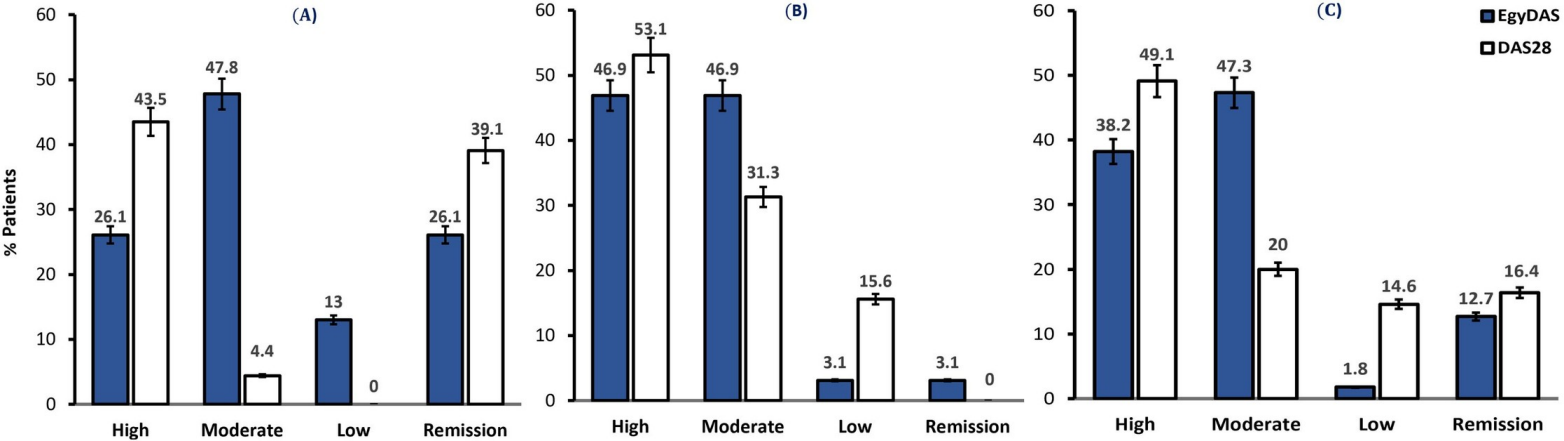
Distribution of rheumatoid arthritis disease activity categories for EgyDAS and DAS28 scores. (**A**) Distribution of the control group of 23 patients. (**B**) Distribution of the ankle involvement group of 32 patients, (**C**) Distribution of all the data of the 55 patients.

The distribution of the 55 RA patients was classified by the two scores (EgyDAS and DAS28) and the percentages were plotted in Fig 1. One can see that DAS28 classified a higher proportion of patients as “High” (53.1% in the ankle group vs. 43.5% in the control group, and 49.1% overall) compared to the EgyDAS (46.9% in the ankle group vs. 26.1% in the control group, and 38.2% overall). On the other hand, the EgyDAS classified a higher proportion of patients with ankle involvement as “Moderate” compared to DAS28 in each group and overall (47.3% of all patients, 47.8% of control group, and 46.9% of ankle group). EgyDAS classified a smaller proportion of patients with ankle involvement as low disease activity compared with the controls (3.1% vs.13%), while the reverse occurs with DAS28 (15.6% vs. 0%). Considering remission both scores show lower remission rates in patients with ankle involvement compared with the controls, nevertheless, DAS28 detects no remission cases in the ankle involvement group.

#### Association between EgyDAS and DAS28 parameters with ankle involvement in RA patients

The two groups (patient group: patients with ankle disease, and control group: patients without ankle disease) were compared in all the parameters: the parameters in the DAS28 and EgyDAS equations, to find which parameters have an impact on the ankle disease. Table 3 shows that all the EgyDAS parameters were significantly higher in the ankle group except for MPV which was higher in the controls and all the EgyDAS parameters have a significant impact on the ankle disease (P-value < 0.05). For the DAS28 parameters, SJC showed insignificant differences between the two groups (P-value > 0.05). Side-by-side boxplots for the parameters of the two scores in the studied groups were illustrated in S1 Fig in S1 File.

**Table 3:** Mean ± standard error of the mean (SE) of the EgyDAS and DAS28 parameters for the control group (patients without ankle involvement, n = 23) and ankle involvement group (n = 32) along with the p-value of the independent samples t-test.

Parameters	Group	Mean ± SE	P-value
**TJC (n)**	Control group	4.96±1.8	0.025*
	Ankle involvement group	8.38±1.1	
**SJC (n)**	Control group	1.57±0.48	0.200
	Ankle involvement group	2.44±0.48	
**VAS (cm)**	Control group	3.37±0.75	0.002**
	Ankle involvement group	6.45±0.61	
**ESR (mm/hr)**	Control group	35.7±4.7	0.000**
	Ankle involvement group	65.7±6.9	
**CRP (mg/L)**	Control group	17.14±2.0	0.015*
	Ankle involvement group	23.22±1.3	
**PDW (%)**	Control group	13.74±0.85	0.045*
	Ankle involvement group	15.84±0.55	
**MPV (fL)**	Control group	8.34±0.24	0.011*
	Ankle involvement group	7.63±0.15	

ESR: erythrocyte sedimentation rate (Westegren), mm/hr.; CRP: C-reactive protein, mg/l; PDW: platelet distribution width,%; MPV: mean platelet volume, fL.; TJC:Tender joint count(0–28);SJC: Swollen joint count(0–28);GH: VAS general health patient(mm).

*Means the difference is significance at 5% and

**means the difference is significance at 1%.

#### Discrimination between the two study groups using EgyDAS and DAS28

The two scores, EgyDAS and DAS28, were compared in terms of their ability to discriminate between the control group and the ankle involvement group using several criteria that were displayed in Table 4. It shows significant differences between the two groups (patients with ankle involvementand patients without) in terms of DAS28 and EgyDAS scores (P-value < 0.01), however, the P-value of EgyDAS is smaller compared to DAS28. The Cohen’s d effect size, Glass’s delts, and Hedges’g show that the EgyDAS score was better than DAS28 in differentiating between the two groups (the higher the value, the better). Also, the two scores were compared by estimating the logistic regression. Table 4 shows that EgyDAS was better than DAS28 (smaller P-value, smaller AIC and BIC values, and higher Odds ratio value). More information about the values represented in Table 4 was provided in the supplementary file, S1 File.

**Table 4:** Several criteria to evaluate the ability of the disease scores (EgyDAS and DAS28) of differentiating between the two study groups (patients with ankle involvement and patients without).

Method	Criteria	EgyDAS	DAS28
**Effect size**	T-test Cohen’s d effect size	0.910	0.769
	Glass’s delta	0.826	0.777
	Hedges’ g	0.927	0.768
**Logistic regression of each score**	P-value of logistic regression	0.005**	0.010*
	Odds ratio	2.510(1.31,4.81)	1.511(1.10,2.07)
	Akaike information criterion (AIC)	67.93	71.39
	Bayesian information criterion (BIC)	71.94	75.41
	Operating characteristic cur (ROC)	0.719	0.739

*Means the difference is significance at 5% and

**means the difference is significance at 1%.

Values of the two scores in the study groups were compared in Table 5. Both EgyDAS and DAS28 scores were significantly different between the two groups. Table 5, considering differences between the two groups, revealed a significant higher score in the ankle involvement group compared with the controls.

**Table 5:** Mean ± standard error of the mean (SE) of the EgyDAS and DAS28 scores for the control group (patients without ankle involvement, n = 23) and ankle involvement group (n = 32) along with the p-value of the independent samples t-test.

Disease score	Group	Mean ± SE	P-value
**EgyDAS**	Control group	0.4369±0.25	0.002**
	Ankle involvement group	1.4291±0.17	
**DAS28**	Control group	3.8652±0.37	0.007**
	Ankle involvement group	5.2497±0.32	

RA: rheumatoid arthritis, SE: standard error,

**means the difference is significance at 1%

**Fig 2:**
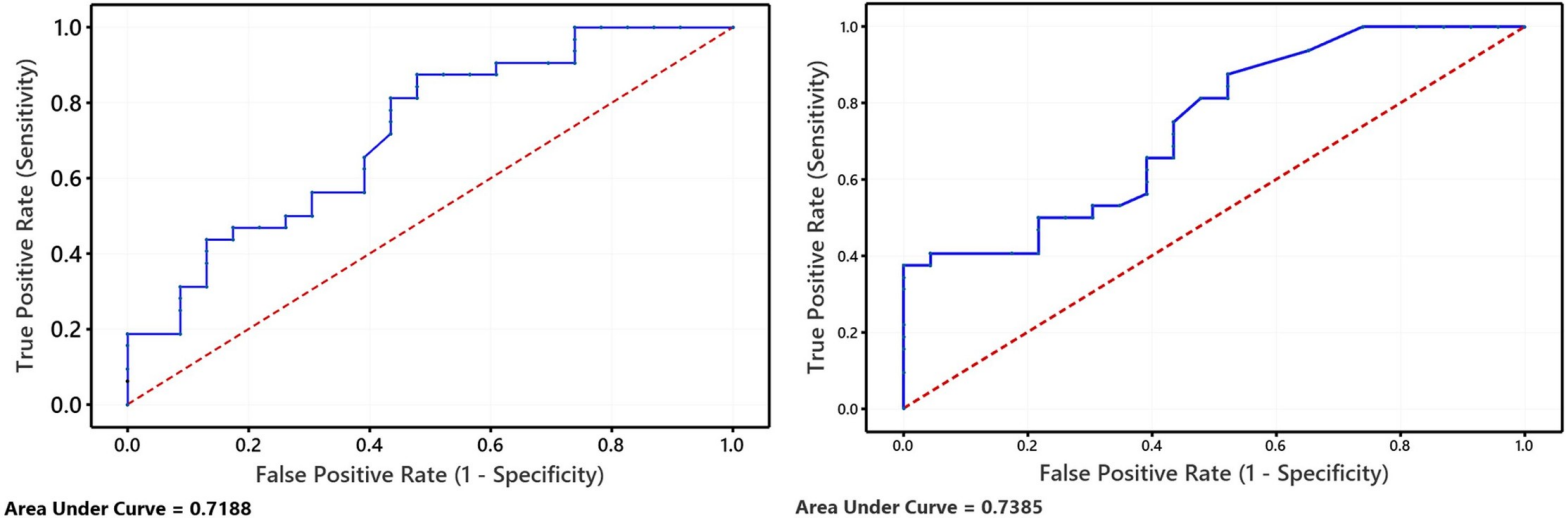
Receiver operating characteristics (ROC) curves of the RA patients in the study. EgyDAS (left) and DAS28 (right).

For the Receiver Operating Characteristics (ROC) curve analysis, they were comparable where the area under the curve (AUC) was 0.719 for EgyDAS and 0.739 for DAS28, both of them were acceptable, as shown in Fig 2.

#### Association between DAS28 and EgyDAS scores in the study groups

**Fig 3:**
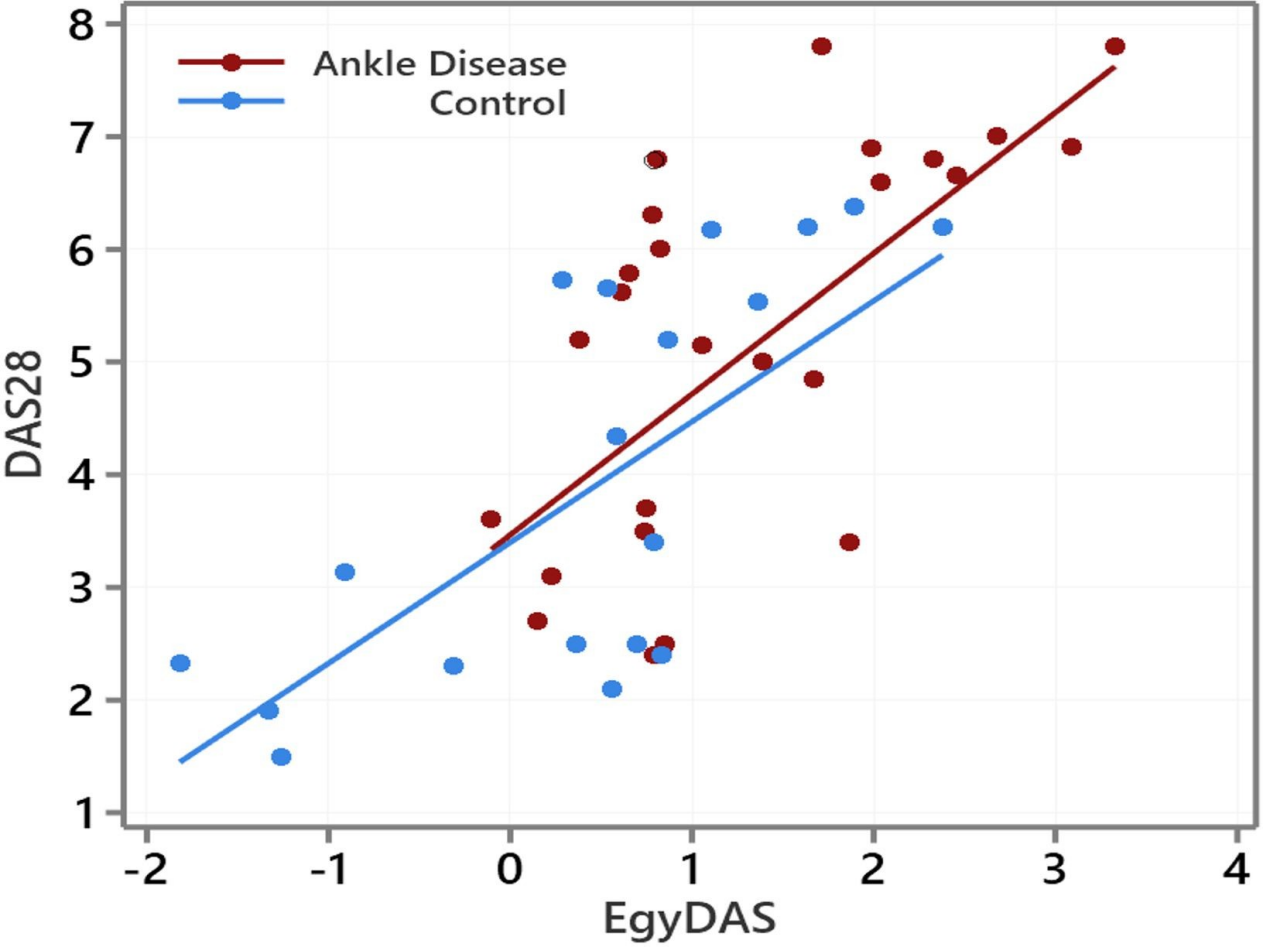
Scatter plot of EgyDAS and DAS28 scores for the two groups: Control and ankle involvement group along with the regression line. Red is for angle involvement group and blue if for the control group.

The two scores (EgyDAS and DAS28) were calculated for all patients (55 patients) and displayed on a scatterplot in Fig 3. It showed that the two scores were associated and the Pearson coefficient of correlation value was 0.732 for all patients. The Pearson coefficient of correlation of the two scores is 0.66 for the ankle disease group, and 0.72 for the control group. Scatterplot by groups of the two scores is displayed in Fig 3.

## Discussion

The role of blood platelets in the pathophysiology of RA was extensively studied, and results revealed that platelets play a central role in the pathogenesis of RA [19, 20]. PIs are a group of hematologic parameters that could be extracted from the complete blood picture and reflect platelets activity. So far PIs include mainly 4 parameters MPV, PDW, PCT, and platelet–large cell ratio (P-LCR). MPV is an indicator of platelet size and has a direct relationship with megakaryocyte numbers. PDW, an indicator of platelet anisocytosis and reflects platelet activation. Thus, it was suggested as a potential diagnostic/prognostic marker in various health problems. P-LCR is another PIs that estimates the percentage of large platelets >12 fL, it has an inverse relationship with platelet count. PCT measures the volume of the blood occupied by platelets, thus estimates platelet mass [21–23].

Automated measurements of plt indices are widely available, cheap, and simple. Almost all automated blood analyzers can determine plt indices with slight variation according to the commercial type of the analyzer [2]. Despite that, there is strong scientific evidence for the clinical significance of PIs in various disease conditions [5, 6], besides the availability and feasibility of their determination. However, there is still a wide gap between availability of PIs and their utility in clinical practice. These facts together with the proven association of PIs with disease activity in patients with RA motivated us to develop a new disease activity score (EgyDAS) for RA, that relies on PIs and other acute phase reactants [15, 16]. This study was conducted to complete the scenario of PIs in disease activity score of RA, by assessing the practicability of the new score in RA patients with ankle involvement. Moreover, the study investigated the validity of EgyDAS in RA patients with and without ankle involvement. To do so, 2 groups of RA patients were recruited one with ankle involvement and the other without. In the current study all joint involvement was thoroughly assessed in each patient not only to detect association with PIs but also to estimate DAS28 which relies on number of swollen and tender joints. Then the value of EgyDAS was calculated for each patient.

Results of this study showed gender and age matching of the two groups, nevertheless, a greater proportion of those with ankle involvement lived in urban areas and was married. These results seem logical as joint involvement in RA correlates with the patient’s physical activity and lifestyle. Clinically, those with ankle involvement have longer morning stiffness and a higher incidence of EAM. Again, these observations were logical as both related to disease activity [24, 25].

The study goes further and assessed the association of ankle involvement in RA patients with hematological parameters. Results revealed that RA patients with ankle involvement showed a higher incidence of anemia and thrombocytosis. Both anemia and thrombocytosis were proved to be associated with a more active form of the disease in RA [15, 26, 27].

Next, we assessed disease activity in the study groups using DAS28 and EgyDAS. Although the findings showed marked discrepancies between EgyDAS and DAS28, it reaffirmed the findings of Khaled et al. [16], in many aspects. One important aspect is the subjective nature of DAS28 compared with the EgyDAS index that results in a higher proportion of patients were categorized as having moderate and high disease activity. In sum, DAS28 overestimates disease activity compared to the new score (EgyDAS). Another aspect is the higher sensitivity of EgyDAS in detecting those in remission and those with low disease activity. Accordingly introducing EgyDAS in treatment decisions of RA patients avoids unnecessary treatment and reduces serious drug side effects.

Then the association between EgyDAS and DAS28 parameters and ankle involvement was explored. The study reported a strong association of ankle involvement and PIs on one hand, and with ESR and CRP in the other hand. The latter was logical where ESR was considered by many investigators a sensitive indicator of the degree of inflammation [28]. Another expected finding, was the insignificant difference of SJC between the two groups, this was because DAS28 neglects foot arthritis [13]. Another astonishing finding was the significant differences in TJC between the two groups, although ankle is not included in TJC as well. Again, these results further proved the subjective nature of DAS28 parameters. Based on these findings EgyDAS would be the most appropriate tool for assessing disease activity in RA patients with ankle involvement. However, the main study limitations were the small sample size of the study, also it would be better to assess PIs and EgyDAS in RA patients with ankle involvement before and after treatment.

This study investigated the ability of EgyDAS and DAS28 to differentiate between RA patients with and without ankle involvement. Results showed that both scores can differentiate between the two groups, however from logistic regression analysis and other criteria EgyDAS was superior to DAS28 in this respect. Finally, the two scores were found associated.

PIs are not commonly used in clinical practice mainly because they are not included in the diagnostic/prognostic workup of many disorders. With the obvious advances in laboratory hematology, we assumed that it is the time to move forward and apply PIs in clinical practice similar to red cell indices.

## Conclusion and recommendations

Conclusively this study proved the value of EgyDAS in assessing disease activity in RA patients with ankle involvement, so as to avoid over or underestimation. This will help tailoring of treatment according to disease activity, thus reducing complications and drug side effects. However, we recommended the use of EgyDAS together with DAS28 to have a full picture of disease activity based on both clinical and laboratory parameters. Moreover, the study raises the definite need for standardization of PIs to ease their extensive use in clinical practice as they are simple, available and relatively cheap disease markers, not only for RA but also for other hematologic and non-hematologic disorders.

## Supporting information

S1 File Additional information about the parameters in Table 4. (DOCX)
