# Supplementary material for: Platelet indices parameters in the new disease activity score of rheumatoid arthritis with ankle involvement: A comparative analytic study
Source: PLoS One. 2021 Sep 16;16(9):e0257200. doi: 10.1371/journal.pone.0257200 (PMC8445486; doi:10.1371/journal.pone.0257200)
Supplement: S1 File — (DOCX) [file pone.0257200.s001.docx]

**Supplementary data of the article:**

**Platelet indices parameters in the new disease activity score of Rheumatoid arthritis with ankle involvement: A comparative analytic study**

**Authors: Safaa A. A. Khaled ^1^,** **Hamdy F. F. Mahmoud^2, 3*^**

^1^Department of Internal Medicine, Clinical Hematology Unit, Assiut University Hospital/Unit of Bone Marrow Transplantation, South Egypt Cancer Institute, Faculty of Medicine, Assiut University, Egypt.

^2^Department of Statistics, Virginia Polytechnic Institute and State University, Blacksburg, VA 24061, USA.

^3^Department of Statistics, Mathematics and Insurance, Faculty of Commerce, Assiut University, Egypt.

*Correspondence to: **Hamdy F. F. Mahmoud** Department of Statistics, Virginia polytechnic institute and state University, Blacksburg, VA 24061, USA Email: [ehamdy@vt.edu](mailto:ehamdy@vt.edu)

**Short title:**  **Platelet Indices in Disease Activity Score of Rheumatoid arthritis**


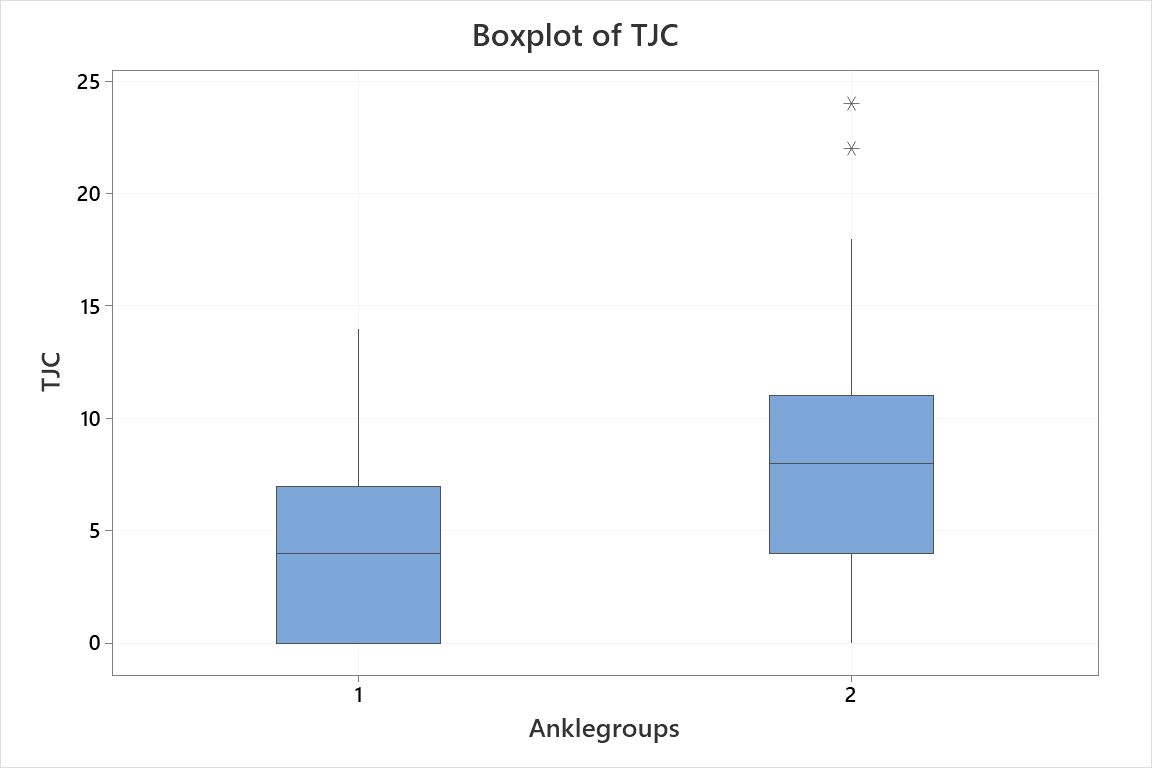

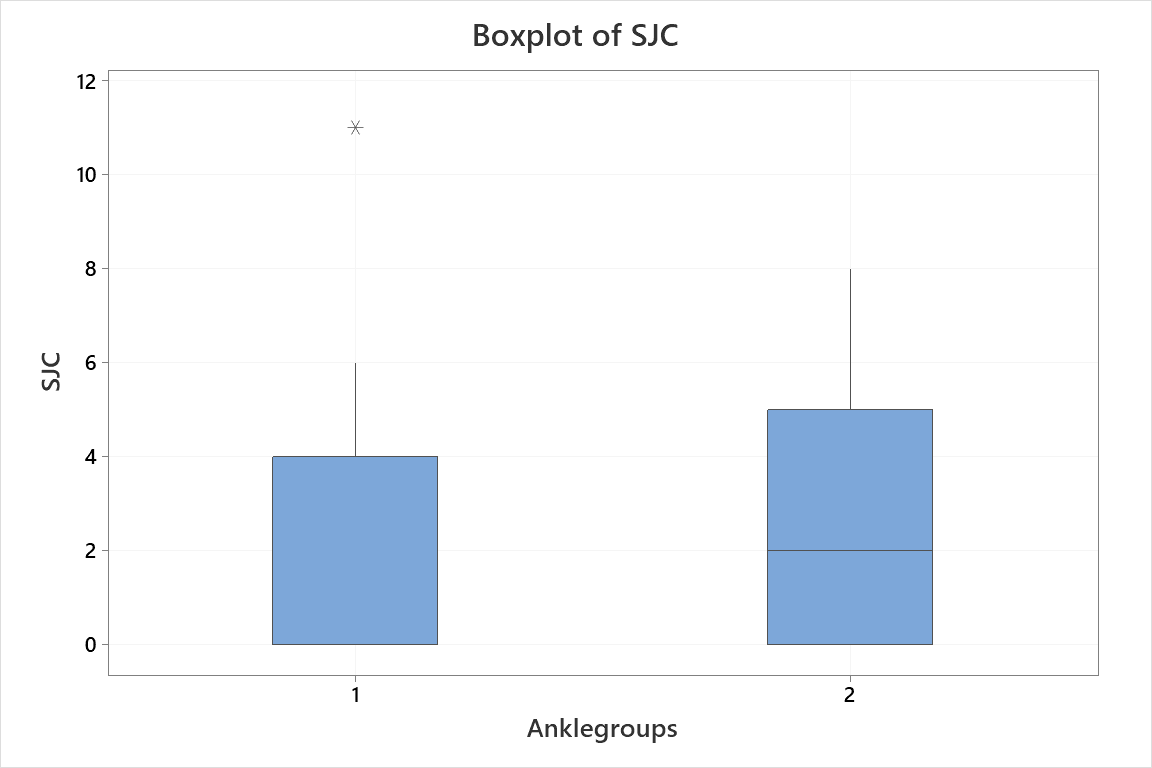


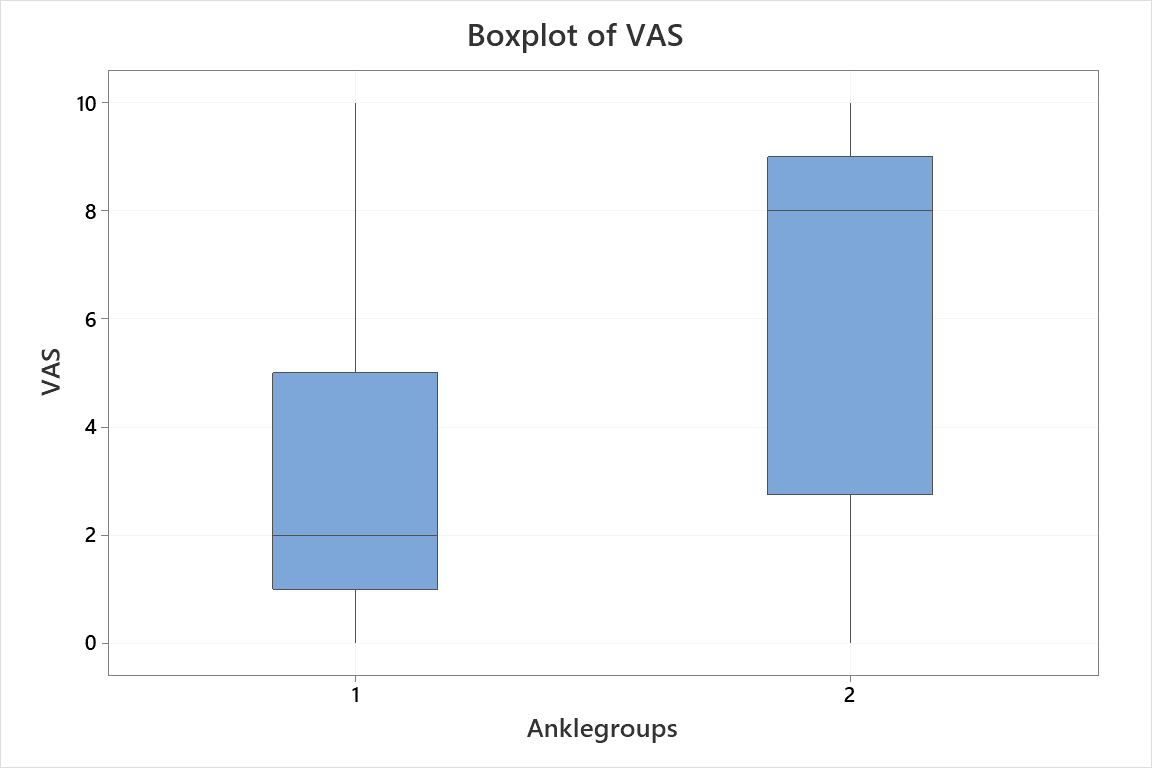

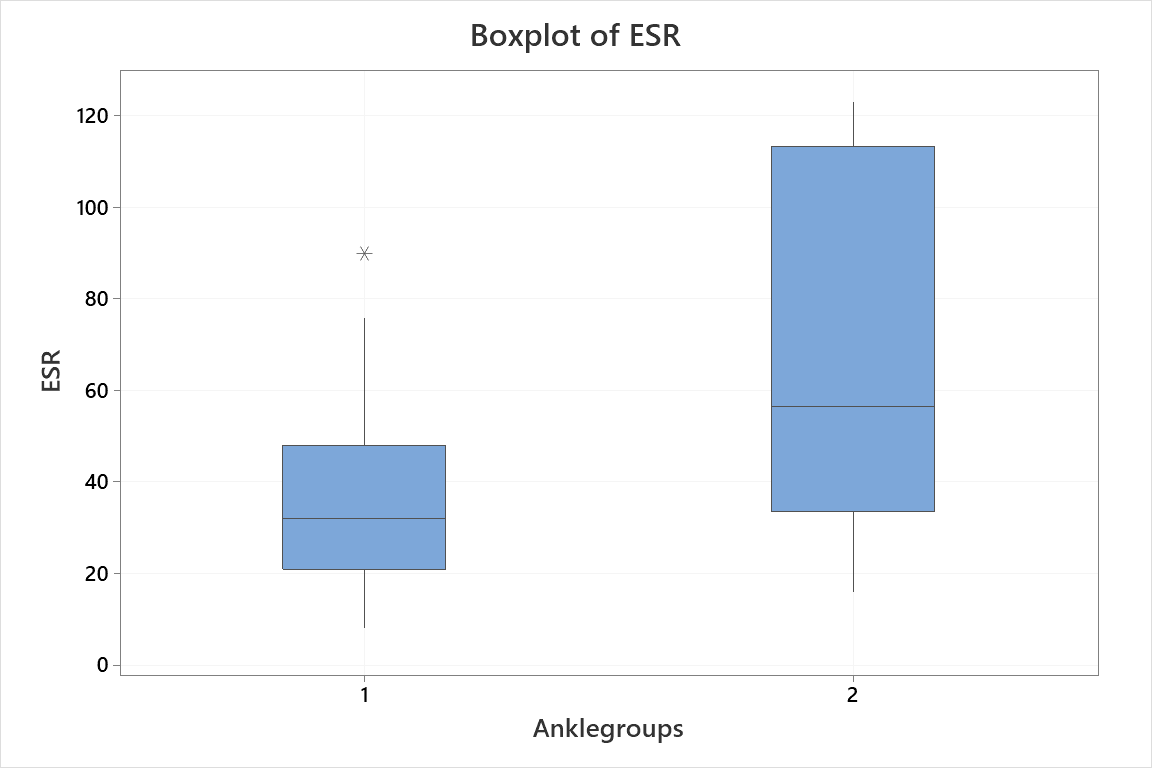


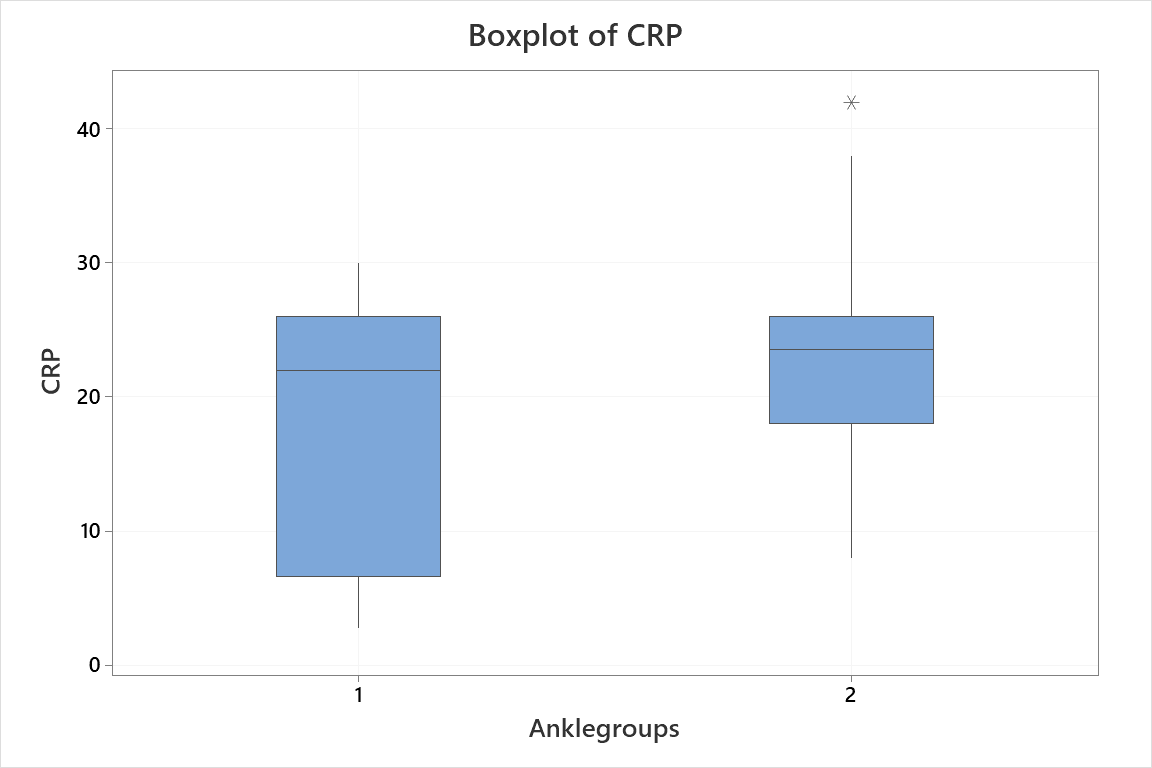

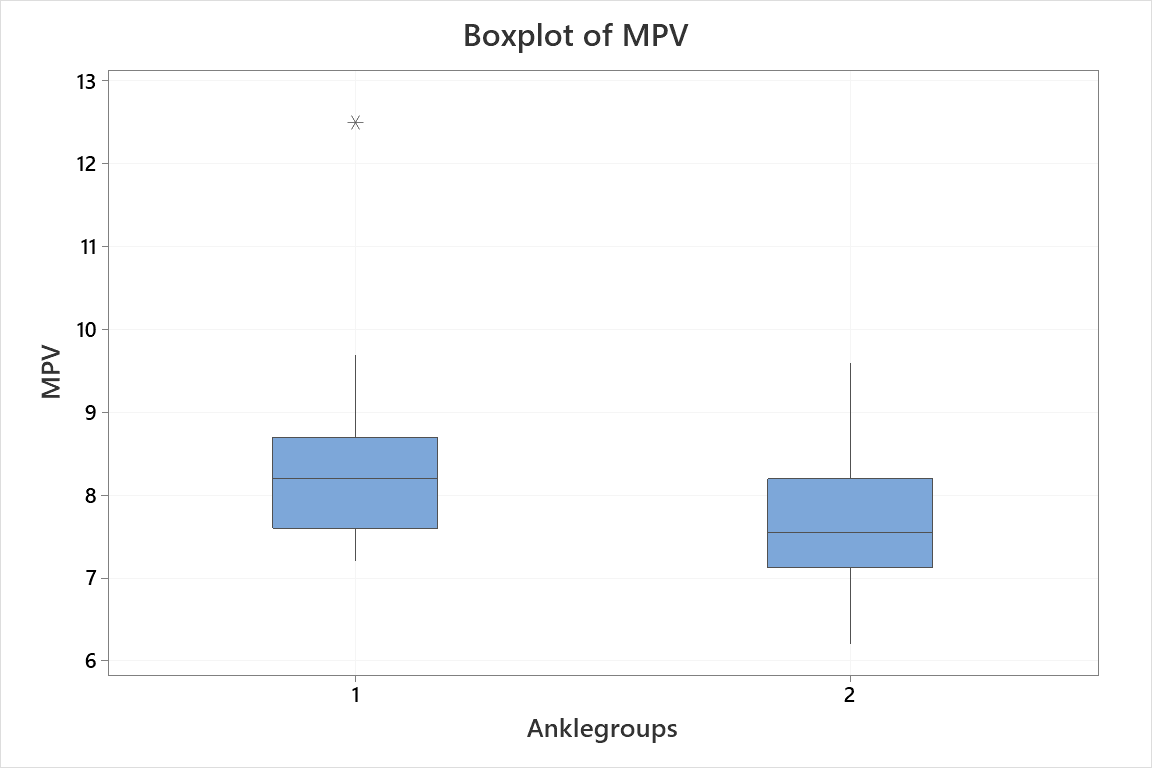


**Fig. Side-by-side boxplots of EgyDAS and DAS28 parameters in the two studied groups , group 1: control and group 2: ankle involvement group.**

**More information about the parameters presented in Table 4**

Cohen's d: it is the appropriate effect size measure if two groups have similar standard deviations and are of the same size.

Glass's delta: it uses only the standard deviation of the control group and is an alternative measure if each group has a different standard deviation.

Hedges' g: it provides a measure of effect size weighted according to the relative size of each sample and it is an alternative where there are different sample sizes.

Cohen (1988) defined the effect size as "small if d = .2," "medium if d = .5," and "large if d = .8".
